# Transparent human – (non-) transparent technology? The Janus-faced call for transparency in AI-based health care technologies

**DOI:** 10.3389/fgene.2022.902960

**Published:** 2022-08-22

**Authors:** Tabea Ott, Peter Dabrock

**Affiliations:** Chair of Systematic Theology II (Ethics), Faculty of Humanities, Social Sciences, and Theology, Friedrich-Alexander-Universität Erlangen-Nürnberg, Erlangen, Germany

**Keywords:** Transparency, AI, Learning Systems, Intelligibility, Health Care, Ethics, Infraethics, Data

## Abstract

The use of Artificial Intelligence and Big Data in health care opens up new opportunities for the measurement of the human. Their application aims not only at gathering more and better data points but also at doing it less invasive. With this change in health care towards its extension to almost all areas of life and its increasing invisibility and opacity, new questions of transparency arise. While the complex human-machine interactions involved in deploying and using AI tend to become non-transparent, the use of these technologies makes the patient seemingly transparent. Papers on the ethical implementation of AI plead for transparency but neglect the factor of the “transparent patient” as intertwined with AI. Transparency in this regard appears to be Janus-faced: The precondition for receiving help - e.g., treatment advice regarding the own health - is to become transparent for the digitized health care system. That is, for instance, to donate data and become visible to the AI and its operators. The paper reflects on this entanglement of transparent patients and (non-) transparent technology. It argues that transparency regarding both AI and humans is not an ethical principle per se but an infraethical concept. Further, it is no sufficient basis for avoiding harm and human dignity violations. Rather, transparency must be enriched by intelligibility following Judith Butler’s use of the term. Intelligibility is understood as an epistemological presupposition for recognition and the ensuing humane treatment. Finally, the paper highlights ways to testify intelligibility in dealing with AI in health care ex ante, ex post, and continuously.

## Introduction

Artificial Intelligence (AI) is an umbrella term for different technologies such as Machine Learning (ML) and Deep Learning (DL) (Iqbal et al., 2021, 11–13). According to the UNESCO, AI systems are “information-processing technologies that integrate models and algorithms that produce a capacity to learn […] leading to outcomes such as prediction and decision-making” (UNESCO, 2022, 10). While they are associated with great hopes for improving the quality of life, they also pose several ethical challenges and require good governance. This is especially important when it comes to health care. AI is expected to be used in nearly all areas of medicine: for improvement in image evaluation and diagnosis finding of different malignancies (Mentzel 2021, 694–704; Aubreville et al., 2019, 67–85; Kashif et al., 2021, 74) up to the detection of stress (Hwang et al., 2018; Oskooei et al., 2021), depression (Uddin et al., 2022, n. p.), and other mental diseases (Lee et al., 2021, 856–864). For the AI to actually improve human diagnosis and treatment, it must be trained with a large amount of non-messy data. These data are categorized as highly sensitive by the GDPR Art. 9. Data relevant for AI-based health care includes not only bodily data but also data collected from daily life. Transactional data from grocery stores, socioeconomic status, education, neighborhood, and physical environment, for example, can become relevant for public health policy (Lu et al., 2020; Artiga and Hinton, 2018, n. p.). These examples show how the measurement of the human and their transparency is extended. At the same time, methods of DL are deployed. This confronts stakeholders with self-learning systems based on a deep neural network with multiple hidden layers (Goswami, 2020, 8–10; Maschewski and Nosthoff, 2021, n. p.). On the one hand, these multiple hidden layers increase the accuracy of a system. On the other hand, they turn the system into a “black box” whose mapping between input and output is no longer comprehensible to the relevant stakeholders (Zerilli et al., 2021, 28–29). Although there are technical approaches to open the black box, questions of modality, execution, and consequences are still open (Lima et al., 2022, 1–18; Arik and Pfister 2019, n. p; Lundberg and Lee, 2017). However, the opaqueness of the AI system is not solely based on the technical complexity of the system. Transparency issues also arise from human-machine interaction within the greater context of a social web of norms, values, and preconceptions that precede and follow the application (Latour 2000). The context of data acquisition, classification (Bowker and Star, 2000, 10–12) as well as the further handling of the output poses challenges for transparency as well. With this change in health care towards its increasing opacity, new questions of transparency arise. Moreover, almost all recent recommendations for governing AI applications cover this topic. Transparency appears as a decisive feature AI should have. This observation provides the starting point of the analysis, which studies the concept of transparency and the assumptions on which the concept is based. As a first step, it should be noted that transparent AI is closely related to the transparency of the people interwoven with it, especially the patients. While the complex human-machine interactions, as well as the AI system itself, tend to become non-transparent, the patient instead becomes seemingly “transparent” by the use of these technologies. Papers on the ethical implementation of AI plead for transparent AI but neglect the factor of the seemingly more and more transparent patient as intertwined with AI. The aim of the paper is to give depth to the concept of transparency and raise awareness for a certain ambiguity. Transparency is “Janus-faced” and can, under certain circumstances, harm human beings and their entitlement to human dignity. Giving more data does not necessarily lead to desired outcomes - e.g., better treatment. The risks and benefits of becoming transparent are not distributed equally among people (Seyyed-Kalantari et al., 2021; Mann and Matzner, 2019; Braun and Hummel, 2022, 4). Obermeyer et al., for example, showed that an AI algorithm perpetuated the systematic inequalities for People of Color. The algorithm identified People of Color as a group with poorer access to care. But instead of changing the situation for the better, the use of the algorithm resulted in less health care spending on Black patients to equally sick White patients (Obermeyer et al., 2019; Röösli et al., 2021, 191). Another example of harmful transparency is the handling of health data of Indigenous people (not only) during the COVID-19 pandemic (Carroll et al., 2019; Carroll et al., 2021). The data collected about Indigenous people is rarely by or for Indigenous people’s purposes (Carroll et al., 2019, 3; Walter, 2018, n. p.). Finally, harmful transparency may result from the connection between the health care system and other economically oriented institutions. In Germany, it is nearly impossible to become a civil servant or to get insured against occupational disability if diagnosed with certain conditions. In a second part, the paper offers a suggestion for coping responsibly with this ambivalence. Transparency will then be presented as an “infraethical” (Floridi, 2017, 391–394) prerequisite that needs to be complemented by the actual ethical notion of intelligibility. Here, intelligibility, following Judith Butler, is vital for the humane treatment of a person. For this reason, transparency in the context of AI should be enriched by the concept of intelligibility. Thereby, the vulnerability of an increasingly transparent patient in the digitized treatment situation can be tackled. Finally, building on the concept of intelligibility, participatory strategies for practice are proposed.

## The claim for transparent AI in current governance recommendations

One of the key principles for governing AI in health care and beyond appears to be transparency. It is one of the most elaborated terms in current governance guidelines (Fjeld et al., 2020, 41; Jobin et al., 2019, 391; UNESCO, 2022; High-Level Expert Group on Artificial Intelligence, 2019). Often, it is mentioned together with explainability or interpretability. This paper follows John Zerilli by distinguishing between transparency as an umbrella term and explainability as one of its subcategories (Zerilli et al., 2021, 25). Explainability and the discourse around explainable AI (XAI), according to Zerilli, is very much concerned with technical transparency - especially the transparency of the algorithm (view also: Lima et al., 2022, 3; ACM US Public Policy Council, 2017; Floridi, 2017, 391–394; Arrietta et al., 2020, 85, 88–90). However, transparency covers more than the understandability of the algorithmic decision-making. It encompasses the social dimension regarding responsibility, accessibility, or justifiability, the role of the patient or physician, and last but not least reflections on social attributions or bias as well. In this paper, the focus lies on the broader and fuzzier concept of transparency. When facing the implementation of transparent AI, several difficulties arise.

First, transparency is an ill-defined term, that is used differently in various contexts. This can be illustrated by the following simple questions, which, despite their straightforward nature, hardly ever receive a clear answer: what is transparency? What is to be made transparent? To whom? To what end? And how is it finally implemented? While the last question concerns practical effects, the first three questions introduce a deeper level of transparency, which is often disregarded in current governance papers. Many of those view transparency as an ethical principle (Fjeld et al., 2020, 41–45; High-Level Expert Group on Artificial Intelligence, 2019, 13, 18; WHO, 2021, 26–28) which, adapted in modules (e.g., open-source data), can be implemented in practice. The questions already show that transparency is about making information available, while leaving open what information, for whom, and for what purpose. However, it is quite clear that making transparent requires different action depending on the addressee. Patients have different know-how and emotional involvement than developers, physicians, or deployers. Accordingly, individual addressees of transparency (transparent to whom?) often go hand in hand with different objectives (transparent to what end?). For instance, making the AI system transparent to a patient is usually associated with the aim of effecting trust (Felzmann et al., 2019, 5; Adams, 2018, 17; Lupton, 2015, 576). In contrast, making the AI system transparent to a developer focuses on efficiency or interoperability (Arrietta et al., 2020, 84; Zerilli et al., 2021, 24; Prabhakaran and Martin, 2020, 72). Finally, in societal or legal contexts transparency aims to sustain accountability (Diakopoulos, 2020, 197) or liability.

Outlining this basic definition problem of transparency leads to a first critical observation: there is no timeless or contextless agenda when making AI transparent. Transparency does not follow an all or nothing logic (Ananny and Crawford, 2018, 979; Zerilli et al., 2022, 7). It always (consciously or unconsciously) excludes crucial information and is highly dependent on its sociotechnical contexts (Hasselbalch, 2021, 10–11; Bowker and Star, 2000, 32). Thereby, transparency is treading a fine line between revealing too much information or too (use)less information. Both ways, revealing too much information and risking an information overflow as well as revealing too less or negligible information, would in the end lead to greater opacity. However, even if the balance succeeds, a remaining opacity stays. This is especially true for the complex sociotechnical process in which an AI is embedded. Not only the interplay between data sets and code yields opaqueness (Burrell, 2016, 5): the interaction of different actants (AI, data, humans) is the decisive factor that favors opacity. Transparency must reflect on these blind spots. It must be marked as a limited process, which is neither free of opacity nor reveals “truth” in any form. As Chesterman puts it: “illusory transparency can be worse than opacity” (Chesterman, 2021, 166).

Another important limitation of transparency is its ethical indifference. Transparency does not necessarily draw consequences from what is disclosed.

On the one hand, transparency does not entail ethical judgement. It does not yet constitute a framework with which to evaluate what has been disclosed. Even if a system is classified as transparent - and it has been shown that “making transparent” is very context-dependent and still contains opaque elements - it is not clear that discriminatory structures will be detected (Bowker and Star, 2000, 44–45). Although there is always bias or discrimination (in the sense of differentiation) attached to AI, some forms are considered harmful while others are not. Moreover, “bias is not simply a feature of data that can be eliminated; it is defined and shaped by much deeper social and organizational forces” (Cho, 2021, 2080). The German General Equal Treatment Act (Allgemeines Gleichbehandlungsgesetz, AGG), for example, provides a classification scheme for detecting harmful bias. It states: “The Act protects people who are discriminated against on the grounds of race or ethnic background, gender, religion or belief, disability, age, or sexual orientation” (Federal Anti-Discrimination Agency, 2019). However, discrimination is not easily detectable. First, bias can have different causes: Real world patterns of health inequality and discrimination, data bias resulting from discriminatory datasets, algorithmic bias due to deployment practices, or application injustice that occurs in the context of use (Leslie et al., 2021, 2). Second, AI can discriminate by proxy. This form of bias is even harder to detect (Calderon et al., 2019, 17). Proxy discrimination means that although protected attributes (e.g., gender or ethnicity) are not mapped in the data set, other characteristics (e.g., membership in a specific Facebook group etc.) can indicate them (Zerilli et al., 2021, 59). These other characteristics, so-called proxies, lead again to disadvantages and stigmatization for the affected individuals (cf. the works of Obermeyer et al., 2019; Prince and Schwarcz, 2020). Third, it gets even more problematic when the AI discriminates against new groups (e.g., pet owners or others), some of which are not at all comprehensible to humans and which are not protected by the AGG or anti-discrimination law (Wachter, 2022). In case two (proxy discrimination) and three (new groups discriminated against) transparency is not sufficient. In these cases, the non-neutral classification system underlying transparency (e.g., the AGG or more subtle forms) does not necessarily protect the people discriminated against (cf. also Bowker and Star, 2000, 319–322; Mann and Matzner, 2019, 5).

On the other hand, transparency is not necessarily associated with power (Ananny and Crawford, 2018, 978). Transparency which pursues the goal of effecting trust does not primarily intend a self-critical analysis of the AI - especially an analysis that is open to revision and aims to bring about change. Thus, if there is no power or will to deal with an AI that has been unmasked as unfair, the concept of transparency loses all its merit as somewhat ethical principle or ideal. In fact, it is ethically indifferent. Often it is economic interests (e.g., insurances) or (historical) power ambivalences that hinder an appropriate response to transparency. One big issue, for example, is the data collection of marginalized groups. Without including them, transparency is likely to become a stigma (cf. Carroll et al., 2019; Wachter and Mittelstadt, 2019). In conclusion, it is misleading to view transparency as an ethical principle, as proclaimed by the current governance guidelines. It is not good per se, like justice, fairness, or non-maleficence, but Janus-faced. Therefore, transparency cannot be set up alongside ethical principles without acknowledging its ambivalence, which arises from its contextualization. This applies particularly to dealing with the permanent remainder of opacity and the handling of “uncovered” injustice.

## Skepticism towards the “transparent patient”

Deeply intertwined with transparent AI is the transparent patient whose health data is the lifeblood of the machine. When it comes to transparency of AI in health care, sociotechnical human-machine interactions are involved. Therefore, to define and specify transparency regarding AI, it is essential to consider the transparency of the humans involved. Primarily, these are the data subjects, i.e., patients. Regarding AI, transparency is seen as a desirable goal, while transparency regarding the patient is rather treated with skepticism (Strotbaum and Reiß, 2017, 367–369; Maschewski and Nosthoff, 2021, n. p.; Prainsack, 2017, 50–51; Pasquale, 2015, 3–4). Here, too, the questions “transparent for whom?” and “transparent to what end?” show the multifaceted nature of transparency. Initially, it is hoped that by collecting large and diverse amounts of an individual’s data, more accurate diagnoses and treatment decisions can be made. Even social or lifestyle data (e.g., a person’s residence, shopping behavior etc.) become relevant (Hague, 2019, 222; Prainsack, 2017, 5–7). Together the various data types form a network of “biomedical big data” (WHO, 2021, 35). The aim is to make a person transparent to enable better diagnosis and treatment.

However, as before, the notion of transparency must be considered as essentially characterized by moments of opacity. The process of making humans transparent in health care is always fragmented. Here, too, classification systems have a significant influence. However, denying the fragmentarity and persistent opacity can lead to serious harm. Transparency is often associated with telling or revealing “the truth” (Ananny and Crawford, 2018, 974). The assumption that “truth is correspondent to, or with, a fact” (David, 2015, n. p.) then could lead to the conclusion that the more facts are revealed, the better the human self can be known (Ananny and Crawford, 2018, 974). In digitized health care, the patient appears as “data body” (Gitelman, 2013, 121). There is a danger that this data body becomes absolute with respect to the data subject: “The data body is the body by which you are judged in society, and the body which dictates your status in the world. What we are witnessing […] is the triumph of representation over being” (Gitelman, 2013, 121). This statement makes clear that our digital representation in health care (and beyond) can gain an ontologically antecedent status. Not solely, but also Christian ethics draws attention to the mysteriousness, and not only puzzling nature of the human being (Jüngel, 2010, 534–536). A human is not the sum of their parts. The reality is more complex than an AI system can describe (Bowker and Star, 2000, 103; Stark, 2014, 94). Therefore, it is also important to consider how the person is embedded in the world in which they live. A diagnosis is preceded by very different notions of a good life, of health and illness etc. For the bodily person, who cannot explain herself entirely, there nevertheless must be the possibility of integrating the AI diagnosis into their narrated and responsive self-perception. It must be clear that the data show a certain part of the person but do not completely remove the opacity of the person - which is not necessarily bad, if seen as a mystery.

The second important aspect is again the ethical indifference of transparency. People give sensitive health data, i. a., with the expectation that it will benefit them. However, to be beneficial, the AI must meet various requirements. For instance, the AI must have been trained with sufficient comparative data from other patients of the same gender, age, disease etc. With lack or underrepresentation of training data of persons with, for example, a certain gender or sexual orientation, “Data Gaps” arise (Criado-Perez, 2019, 217–235; Norris et al., 2020, 2; Hatzenbuehler and Pachankis, 2021, 437; Dankwa-Mullan et al., 2021, 223–224). This can lead to poorer or even erroneous diagnoses and treatment decisions. For this reason, it bears greater risk for some people, especially minorities, to become transparent than for others. The problem gets even more intense when we consider the phenomenon of intersectional discrimination. A person can face discrimination not only on one but on the intersection of several characteristics. Kimberlé Crenshaw makes this particularly explicit regarding the intersection of gender and race. She claims that antidiscrimination measures overlook people standing at the crossroads of discrimination, namely Black women (Crenshaw, 1989, 140, 149). However, intersectional discrimination can involve other factors as well. Which characteristic or which concurrence of different characteristics (obesity, disability, habits etc.) leads to stigmatization is not clear from the outset as these markers not necessarily appear in the analyzed data. Though, what shows up in the data are proxies. At a first glance, they do not appear as stigmata. For example, living in a certain neighborhood can function as a proxy (Prince and Schwarcz, 2020). Therefore, some people are skeptical about becoming transparent when providing data, for good reason. They are more likely to face increased vulnerability or precarity (Carroll et al., 2019; Butler, 2009, 25). This is due to the fact that there is no response to their transparency - first, on a diagnosis and treatment level, second, on a societal level (e.g., disadvantage in insurance). The data collection on Indigenous people in the United States illustrates this point clearly (Carroll et al., 2019, 3). Although transparency can be damaging to people, it can also bring them into focus and mobilize resources to address their situation (Casper and Moore, 2009, 79). Some may consider this a chicken-or-egg question: without transparency, there will be no better treatment and diagnoses in the future. Vice versa, if there is no prospect of getting good treatment, transparency will be experienced as harmful. Therefore, the paper aims to enrich the actual claim for transparency by a critical societal perspective. Transparency is not an ethical principle per se. A deeper philosophical analysis is needed to portray transparency as Janus faced and, one could say, “infraethical” (Floridi, 2017, 391–394) term.

## Transparency as a Janus-faced infraethical concept

It is rightly pointed out that the demand for transparency initially sounds like a desirable ideal. Its status as an “inherent normative good” is often associated with other values such as truth-telling, honesty, or straightforwardness (Viola and Laidler, 2021, 23). Additionally, transparency is often misunderstood as revealing or showing the truth of something. Regarding AI applications, transparency is treated as “a panacea for ethical issues” (Mittelstadt et al., 2016, 6). However, transparency is not enough to address unfairness, discrimination, and opacity (Edwards and Veale, 2017, 21–22). The Janus-faced character of transparency becomes especially evident when considering, first, the remaining opacity and, second, the not necessarily given connection with awareness of injustice and the power to do something about it. As for the first point, the process of making transparent runs the risk of neglecting the veil that is lifted at that very moment (Kilian, 2013, n. p.). If the different filters (Who? What? To Whom? With what aim?), that determine to what extent the veil is lifted, are blanked out, transparency runs the risk of working as an illusion (Adams, 2018, 17). Regarding the second aspect, the only loose connection between transparency and awareness of malpractice or power to change may even threaten human dignity. If the question “Transparent to what end?” is answered with “To build trust” (concerning AI) or “To make visible for the health care system” (concerning humans) is not enriched by a watchful function against instrumentalization, it is misled and again cherishes an illusion.

**FIGURE 1:**
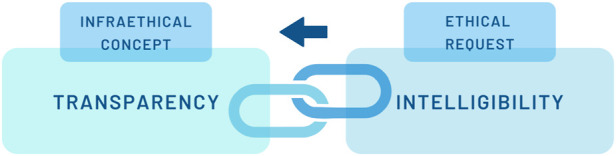
Intelligibility as ethical request.

Finally, this in-depth analysis of transparency as Janus-faced leads to the conclusion that transparency is not an ethical principle per se but an “infraethical” (Floridi, 2017, 391–394) concept. Infraethical means that it is a “not-yet-ethical framework of implicit expectations, attitudes and practices that can facilitate and promote moral decisions and actions” (Floridi, 2017, 192). Thus, regarding the learning system, transparency can build the ground for awareness of malpractice. As for the patient, it is necessary to give as much information as possible to get a chance for better diagnoses and treatment. However, as Floridi puts it: an injustice regime can be transparent, too, without being for this any less evil (Floridi, 2017, 393). To just apply infraethical transparency to foster successful facilitations (e.g., build trust, implement the technique easier, etc.) is not enough protection of human dignity. Rather, what Floridi suggests is that the infraethics must be combined with “morally good values (the right axiology)” (Floridi, 2017, 393) and be shaped by them. In the following, this reminder of Floridi will be taken as a basis. While Floridi primarily refers to transparency in relation to the design of AI, this view will be enriched by the previous investigations on the transparent human. With the focus on the human, a social anthropological perspective challenges the infraethical concept of transparency. It refers to the need for intelligibility, which can be made a critical requirement for transparency claims (cf. Figure 1). In demanding intelligibility as a verification framework for transparent humans in digitized health care, the identified obstacles of transparency will be tackled: That is first, non-reflected opacity, and second, ethical indifference from not recognizing harm and/or lack of agency.

## How to avoid increased vulnerability caused by transparency? Using intelligibility as an ethical request

**FIGURE 2:**
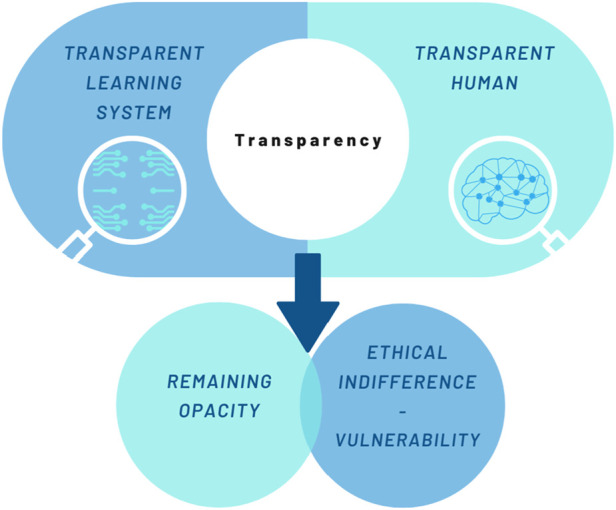
Characteristics of transparency.

The previous section has shown that transparency is a Janus-faced concept. Its positive or negative impact on an individual is highly contextual and is often driven by a socio-historical or political agenda. Behind this is the idea that “making transparent”, firstly, is itself a highly difficult and elusive process of negotiation between humans and the system. It always contains elements of opacity. Secondly, transparency does not yet produce an appropriate response to the exposure. Rather, it is ethically indifferent and can lead to increased vulnerability (cf. Figure 2). Having discussed the ambivalence of transparency, the final section of this paper addresses ways in which transparency can be reframed. The section moves on to describe how to avoid the possible negative effects of human transparency (increased vulnerability, stigma, or harm). Further, it offers a way to address unfairness, discrimination, and opacity in the context of transparent AI. For this purpose, the paper suggests enriching transparency with intelligibility. The term intelligibility is used here in accordance with Judith Butler. Butler uses it when she discusses what precedes personhood. She asks for a “new bodily ontology” in order to rethink “precariousness, vulnerability, injurability, interdependency, exposure, bodily persistence, desire, work and the claims of language and social belonging” (Butler, 2009, 2). Following Hegel, she assumes that humans are necessarily dependent on structures of recognition (Butler, 2009, 2–3). However, these structures of recognition are shaped by norms and classifications. Butler refers to norms as something that operates “to produce certain subjects as ‘recognizable’ persons and to make others decidedly more difficult to recognize” (Butler, 2009, 6). Consequently, the norms applied have an impact on individual vulnerability or precarity (Butler, 2009, 25). A deeper understanding is provided by Butler’s distinction between apprehension and intelligibility. In *Frames of War* Butler defines apprehension as the “knowing that is not yet recognition” (Butler, 2009, 6). Intelligibility, on the other hand, is described as a “general historical schema or schemas that establish domains of the knowable” (Butler, 2009, 6). Butler exemplifies this with the category of gender, which is shaped by the schema of heteronormativity (Butler, 2007, 23–24). Further, Butler notes that intelligibility builds the ground for norms of recognizability. These norms of recognizability in turn prepare the way for recognition (Butler, 2009, 6). In summary, intelligibility is the foundation of the discourse of humans speaking as humans and not “as-if-humans” (Butler, 2004, p 30). Therein, it differs from transparency (and apprehension). Intelligibility is about something preceding (and at the same time following) the visible. In order to follow this ontological description, a distinction between the terms “to perceive” and “to recognize” may be helpful. While perceiving, on the one hand, only grasps the cognitive identification, recognizing, on the other hand, is part of an evaluative acknowledgment (Honneth, 2003, 26–29). The latter reaches to the very roots of being human: to recognize someone means to acknowledge someone as human and therefore as an addressee of human dignity. The concept of intelligibility, according to Butler, offers an explanation for how identities are constructed within normative practices (Halsema, 2005, 216). This way, human dignity violations can be detected. The presupposition of being recognized as a human is to be intelligible as a human. Intelligibility, understood this way, is circumscribed in existing norms. Norms can relate to sex, gender, desire, and race, for example. This observation is of great importance when it comes to AI. In a particular way, the classification and pattern recognition that constitutes AI shows that the technology is embedded in social norms and values (Jasanoff, 2016, 266).

Now, what does this mean for transparency?

First, transparency without the request for intelligibility can lead to the invisibility of a person. This phenomenon is covered in Alex Honneth’s essay collection *Unsichtbarkeit. Stationen einer Theorie der Intersubjektivität* (*Invisibility. Stations of a Theory of Intersubjectivity*) where he describes invisibility as “looking through” a person (Honneth, 2003, 11). This form of disregard can be observed when significant characteristics of a person are not well represented in the training data of an AI, but the AI is still applied to that person. It is exceedingly likely that poorer or no diagnosis or treatment outcomes will be achieved. However, one can argue that transparency tackles exactly this problem: it reveals training data to prevent bias. This is certainly true. But the process of making transparent is also subject to norms and classifications - such as anti-discrimination law. As soon as bias by proxy, intersectional discrimination, wrongful classification (Brindaalakshmi, 2021, n. p.), or completely new - sometimes for humans not even understandable - groups (Wachter, 2022) are affected, transparency does not necessarily benefit the persons affected. All four of these forms of discrimination cannot be identified through the application of existing norms. It needs the question of intelligibility to address these shortcomings of transparency.

Second, transparency that neglects intelligibility can lead to exposure of the human behind the data. If transparency leads to visibility, but visibility leads to social disadvantages, transparency can increase vulnerability. The data collection of Indigenous people (Carroll et al., 2019; Carroll et al., 2021) or Non-Binary people (Brindaalakshmi, 2021) illustrate this point clearly. Without receiving (medical) help or recognition, the exposure is stigmatization. It is perception without recognizing. Therefore, it can be argued that remaining non-transparent can be an advantage since transparency could involve experiencing violence. Becoming transparent can mean being subjected to a norm that is experienced as coercive: this applies especially to those people who do not fit in gender, body, or other group schemata - for people that defy classification.

Although Butler does not use the term intelligibility in an ethical sense, it nevertheless can build the starting point for ethical considerations. Beginning ethical consideration in the perspective of intelligibility questions the fundamentals of the human. It shows the necessity of keeping the notion of the human open to future articulation: “The nonviolent response lives with its unknowingness about the Other in the face of the Other” (Butler, 2004, 35–36). The subject itself is the starting point of the critical evaluation. Their life calls into question the frames which constitute the ontological field (Butler, 2009, 7). Butler considers the deviation from the norm as a potential disruption of the regulatory process that the norm constitutes (Butler, 2004, 52). This norm can be societal (e.g., gender), technological (e.g., due to non-representative data training), or sociotechnical (a combination of both). Some lives exist between, outside, or across the norm. They make a demand on the existing framework, revealing the shifting character of the grids of intelligibility. To detect the disruptive potential of those lives and to make use of it for improving AI is a future challenge. In this regard making transparent is like scratching the surface of the black box to make just a small detail visible. This visibility then has to put up with the critical inquiry of intelligibility. Transparency itself is not a changing force, but it gives hope that sensitivity for intelligibility can make transparency “better”, e.g., through iterative transparency with, first, simultaneous knowledge of the opacity due to human-machine interaction and, second, the epistemological power of intelligibility. The challenge to be met is to establish intelligibility as a critical corrective for transparency. It focuses on the human, who is reliant on recognition to uphold human dignity. These considerations will be specified in the following with respect to the transparent human and, finally, derived from this, also for transparent systems.

Now, what is gained by introducing and supplementing the concept of transparency with intelligibility? The paper suggests to make the ethical test criterion for transparent AI the intelligible, i.e., recognizable/acknowledgeable human or patient. Where people are transparent but non-intelligible, as illustrated before with the examples of bias, intersectional discrimination, bias by proxy, discrimination of new and non-protected groups (Wachter, 2022), or data collection of marginalized groups, the existing frameworks become questionable. Intelligibility helps to uncover the “historical *a priori*” (Foucault, 1972, 126–128) in which the AI is embedded. In this regard, critical social analysis can provide starting points for the evaluation of AI and their outcomes. While transparency often follows an all or nothing logic, the term intelligibility opens the opportunity to uncover the essential elements of an AI system: does the system provide an adequate basis for rendering people intelligible? And does it do so not only ex ante during data collection and algorithm design but also continuously during implementation and adaptation, and finally ex post after the actual use case? Further asked: is a person’s condition not only disclosed, but is it responded to appropriately in a medical decision-making situation? The response is the pivotal element intelligibility aims at. Paradoxically, it demands a question as an answer. “Who are you?” is the non-violent response to a human made transparent by AI systems. This question acknowledges the “clipping”-character of personhood. It allows the transparent patient to enter an exchange with the transparent AI, which cannot maintain its objectivity claim. Whether a person is intelligible is not possible to tell only from the outside. Thus, AI must be considered in a personal context of life. This contextualization is relevant for all types of AI. It leads, if necessary, to an extension of “grids of intelligibility” (Stark, 2014, 94). Thus, AI systems are tied back to social conditions and vulnerabilities. “The necessity of keeping our notion of the human open to a future articulation is essential to the project of international human rights discourse and politics” (Butler, 2004, 36). Intelligibility draws attention to the frames and norms transparent AI constructs. It challenges the process of making transparent to reveal the conditions of the foundations of being a person. Hence, the claim of intelligibility incorporates sensitivity to socio-historical and political power structures into measures of transparency (Mann and Matzner, 2019, 7).

## Conclusion: A space for testifying intelligibility

**FIGURE 3:**
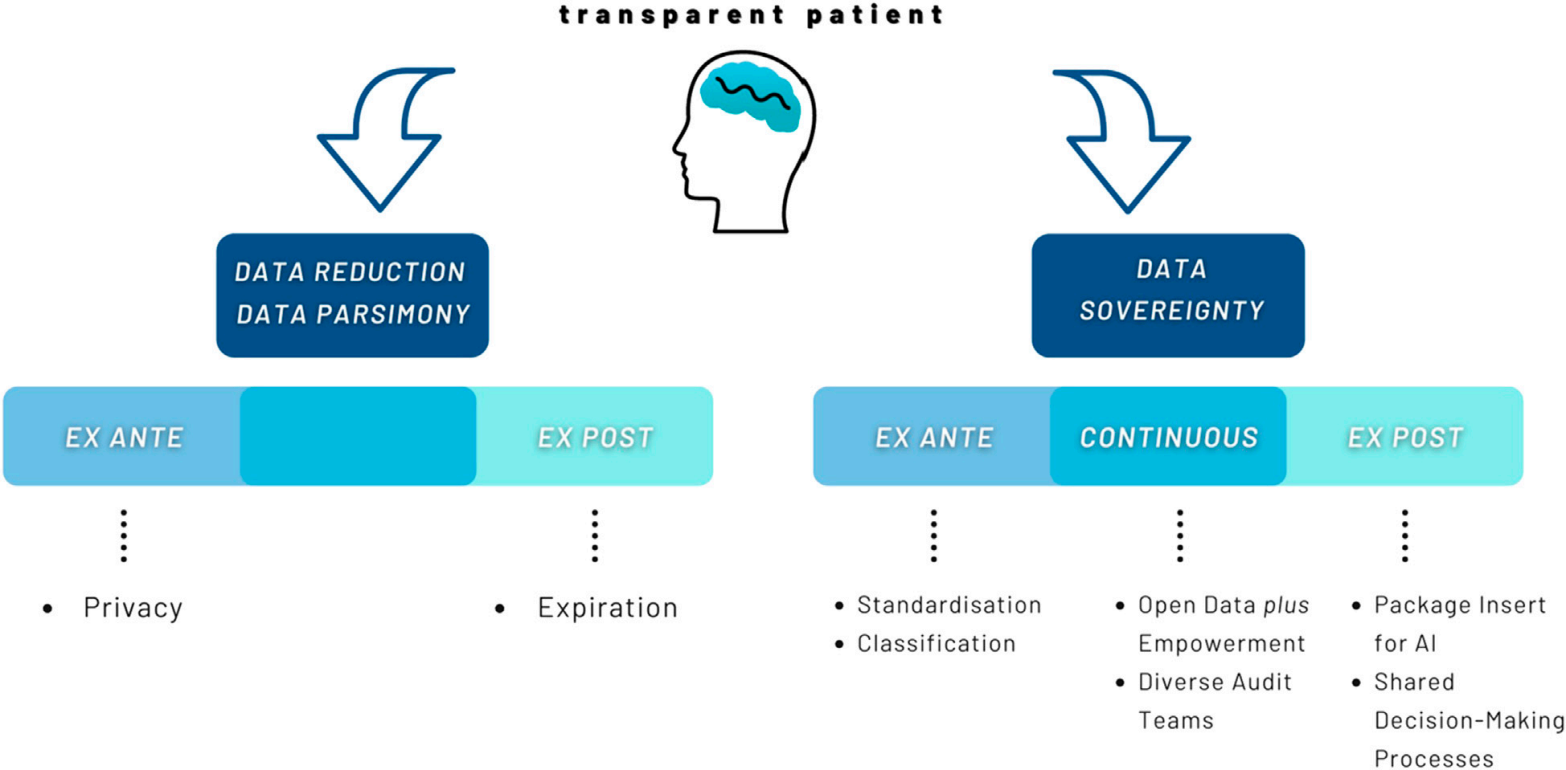
Data reduction vs. Data sovereignty.

Finally, it must be asked what transparency looks like that takes the vulnerability of the people involved seriously. Or even more specific: how to generate attention for frames of intelligibility in digitized health care environments? Further, how can this attention then lead to actual changes regarding non-harmful transparency of humans and AI? Typically, two lines of perspectives prevail in the governance of AI regarding the transparent patient (cf. Figure 3): the first shall be referred to here as the *data reduction* or *data parsimony perspectives*. They focus on the right to refuse provision of data. More precisely: a person needs to be sovereign in terms of the information she wants to give right at the beginning - i.e., ex ante. These perspectives often view organizations as surveillance organisms that misuse data or use humans as laboratory animals (Véliz, 2020, 39, 65). Their result is to give no or hardly any data at all or erase it as soon as possible (Mayer-Schönberger, 2009, 171–173) – i.e., ex post. This would, in a sense, lead to conscious and intentional hazarding of the consequences of a person’s “non-intelligibility”. Considering an increasingly digitized health care system and the benefits that AI offers in terms of diagnosis and treatment, not giving data would lead to health care disadvantages and inequality. Thus, non-intelligibility will not be tackled by giving no data. It rather will exacerbate inequalities and further increase societal problems.

The second line are to be referred to here as *data sovereignty perspectives*. They focus on the development process of AI as well as the outcomes of its use, i.e., ex ante, continuous, or ex post. Behind this is the conviction that not giving data is not an adequate solution to solve problems of (non-)intelligibility and thus violations of human dignity. Instead, data sovereignty perspectives try to deal with the data and suggest solutions on different levels (Hummel et al., 2021b, 22; Hummel et al., 2021a, 9–10; Wachter and Mittelstadt, 2019, 4–5, 13–14). While for data sovereignty perspectives non-intelligibility is not acceptable, the process of making intelligible must likewise meet certain standards in order to not be experienced as violent. Making intelligible goes beyond making transparent. It is sensible to the mysteriousness of the person and their right to be involved in meaning making processes around herself. Further, attention towards frames of intelligibility absorbs the digital exposure and endows it with recognition of harm and agency to address it. The awareness of the need for considering intelligibility as an ethical request for transparency leads to the persons affected first. The humans themselves are the stumbling blocks when it comes to detecting discrimination or stigmatization. Their life in relation to the frames of intelligibility brings forward questions and demands for AI. The patient must be given space for a “discourse of self-reporting and self-understanding” (Butler, 2004, 67).

This comes with several implications regarding the data collection and training process: first, if one fears to experience harm during the process of making intelligible, these fears must be taken seriously. In order to address this concern, spaces must be created in which non-intelligibility or transparency is brought up for discussion. Moreover, non-intelligibility must be the critical trigger point to change the system, in which it is better for people to take on health risks than to become transparent but non-intelligible.

Second, the data that are actually collected have to be standardized. Being aware of the issue of intersectional discrimination could mean involving patients to “capture their characteristics in a way that facilitates readability and interoperability” (Norori et al., 2021, 4). In the case of the Indigenous data collection with no purpose for the people concerned it could mean investing in community controlled data infrastructures (Carroll et al., 2021, 4). On the one hand, this could ease the verification of the algorithm in the individual treatment situation. On the other hand, it contributes to data sovereignty at a very early stage. However, some thinkers conclude that protected attributes, like gender or ethnicity, should not be collected or classified at any rate (Zerilli et al., 2021, 59). An intelligibility-based approach to AI must reject this anti-classification approach. Rather, it pleads for a use case sensitive procedure that later discloses its modus operandi. This is due to the fact that in health care it is nearly impossible to exclude sensitive information. Often, these attributes appear by proxy and their discriminatory potential is much more difficult to detect afterwards. Also, it is impossible to perceive causal relations between discrepant factors if these are not collected (Ruf and Detyniecki, 2021, 19). Yet, the hope is to gain error-free results independent of a person’s group affiliation.

Third, many papers mention the need for Open Data. Open Data and Open Science approaches focus on opening up the development process for people to interfere (Huston et al., 2019, 254). The idea behind this is that “if everything is disclosed, everyone has maximum control”. However, several Open Data projects realize that “transparency [alone, authors] is insufficient - a data dump on a portal is not meaningful without sufficient awareness, education, and participation. The same principle applies to algorithms” (Turek, 2020, n. p.). It is not sufficient to only open up the data to the public. The opening process must be supplemented at the same time with opportunities for actual interaction and participation. A study by Schütz et al. shows that people are willing to interact and shape the technologies of the future (Schütz et al., 2019, 137). This goes far beyond transparency and simply being informed (Schütz et al., 2019, 137). The aim must be to enable a diverse set of people to actually check the data sets and to implement heterogenous audit teams. This empowerment of people (e.g., technical literacy, work environments etc.) must be corresponded to by the learning system. The algorithm must, for instance, enable (fast) frame adaptation processes. This is to meet the shifting “grids of intelligibility” and the need to integrate different voices which have not been recognized before. Nevertheless, as the open “debug” competition of Twitter’s cropping Algorithm showed (Meunier et al., 2021, n. p.): datasets will not be free from bias nor is it possible to avoid bias completely at further processing stages. The reason for this is that bias is not necessarily caused by the technological component, the code, or the individual use case. It has a socio-historical dimension of discrimination as well (Meunier et al., 2021, n. p.). Therefore, an ex post security mechanism must be implemented that still allows individuals to request their intelligibility or object to their non-intelligibility in the use case. To identify whether the algorithm actually renders humans intelligible can be accompanied by a kind of “package insert” of a learning system. With a package insert for algorithms, an independent and diverse audit team could provide information about the development process and the nature of the training data. This information must be consciously considered within the shared decision-making process between patient and physician. Thus, the package insert functions as a safety or bias warning to avoid harm. It contributes to drawing attention to frames of intelligibility. By being alerted to which groups of people the algorithm produces worse results for, the medical professional can flexibly adjust her decisions. However, not only the medical professional but also the patient should be informed about this package insert in shared decision-making processes. In summary, transparency regarding AI and humans, enriched by the ethical request of intelligibility, demands to make the individual life courses audible. This is to tackle the persistent opacity of humans as well as of AI. Therefore, participatory approaches become important when practical implementation is concerned. This is implied in Bowker and Star’s proposal for “a mixture of formal and folk classifications that are used sensibly in the context of people’s lives” (Bowker and Star, 2000, 32). Additionally, the learning system must always be open for interference and revision. The shifting grids of intelligibility in everyday life must be representable in the algorithm. That means: the learning system has never finished learning.

## Data Availability

The original contributions presented in the study are included in the article/Supplementary Material, further inquiries can be directed to the corresponding author.

## References

[B1] ACM US Public Policy Council (2017). Statement on algorithmic transparency and accountability. Commun. ACM. Available at: https://www.acm.org/binaries/content/assets/public-policy/2017_joint_statement_algorithms.pdf Accessed Jully 27, 2022.

[B2] Adams R. (2018). The illusion of transparency: Neoliberalism, depoliticisation and information as commodity. SSRN J. 10.2139/ssrn.3281074

[B3] Ananny M. Crawford K. (2018). Seeing without knowing: Limitations of the transparency ideal and its application to algorithmic accountability. New Media & Soc. 20 (3), 973–989. 10.1177/1461444816676645

[B4] Arik S. O. Pfister T. (2019). TabNet: Attentive interpretable tabular learning. Available at: https://arxiv.org/abs/1908.07442 (Accessed May 27, 2022).

[B5] Arrietta A. B. Díaz-Rodríguez N. Del Ser J. Bennetot A. Tabik S. Barbado A. (2020). Explainable Artificial Intelligence (XAI): Concepts, taxonomies, opportunities and challenges toward responsible AI. Inf. Fusion 58, 82–115. 10.1016/j.inffus.2019.12.012

[B6] Artiga S. Hinton E. (2018). Beyond health care: The role of social determinants in promoting health and health equity. Available at: https://www.kff.org/racial-equity-and-health-policy/issue-brief/beyond-health-care-the-role-of-social-determinants-in-promoting-health-and-health-equity/ (Accessed May 30, 2022).

[B7] Aubreville M. Goncalves M. Knipfer C. Oetter N. Würfl T. Neumann H. (2019). in Transferability of deep learning algorithms for malignancy detection in confocal laser endomicroscopy images from different anatomical locations of the upper gastrointestinal tract” in biomedical engineering systems and technologies. Editors Cliquet A. Wiebe S. Anderson P. Saggio G. Zwiggelaar R. Gamboa H. (Cham: Springer International Publishing), 67–85.

[B8] Bowker G. C. Star S. L. (2000). Sorting things out. Classification and its consequences. Massachusetts: MIT Press.

[B9] Braun M. Hummel P. (2022). Data justice and data solidarity. Patterns 3 (3), 1–8. 10.1016/j.patter.2021.100427 PMC9058839 35510188

[B11] Brindaalakshmi K. (2021). A New AI Lexicon: Gender. Transgender erasure in AI: Binary gender data redefining ‘gender’ in data systems. Available at: https://medium.com/a-new-ai-lexicon/a-new-ai-lexicon-gender-b36573e87bdc (Accessed June 4, 2022).

[B12] Burrell J. (2016). How the machine ‘thinks’: Understanding opacity in machine learning algorithms. Big Data & Soc. 3 (1), 205395171562251. 10.1177/2053951715622512

[B13] Butler J. (2009). Frames of war. London, New York: Verso.

[B14] Butler J. (2007). Gender trouble. New York: Routledge.

[B15] Butler J. (2004). Undoing gender. New York: Routledge.

[B16] Calderon A. Taber D. Qu H. Wen J. (2019). AI blindspot. Cambridge: Cambridge University Press.

[B17] Carroll S. R. Akee R. Chung P. Cormack D. Kukutai T. Lovett R. (2021). Indigenous peoples’ data during COVID-19: From external to internal. Front. Sociol. 6, 617895. 10.3389/fsoc.2021.617895 33869569 PMC8022638

[B18] Carroll S. R. Rodriguez-Lonebear D. Martinez A. (2019). Indigenous data governance: Strategies from United States native Nations. Data Sci. J. 18 (31), 31–15. 10.5334/dsj-2019-031 34764990 PMC8580324

[B19] Casper M. J. Moore L. J. (2009). Missing bodies: The politics of visibility. New York: New York University Press.

[B20] Chesterman S. (2021). We, the robots? Regulating artificial intelligence and the limits of law. Cambridge: Cambridge University Press.

[B21] Cho M. K. (2021). Rising to the challenge of bias in health care AI. Nat. Med. 27 (12), 2079–2081. 10.1038/s41591-021-01577-2 34893774 PMC11017306

[B22] Crenshaw K. (1989). Demarginalizing the intersection of race and sex: A black feminist critique of antidiscrimination doctrine, feminist theory and antiracist politics. University of Chicago Legal Forum, 139–167.

[B23] Criado-Perez C. (2019). Invisible women. Data bias in a world designed for men. New York: Abrams Press.

[B24] Dankwa-Mullan I. Zhang X. Le P. Riley W. T. (2021). “Applications of big data science and analytic techniques for health disparities research,” in The science of health disparities research. Editors Dankwa-Mullan I. Pérez-Stable E. J. Gardner K. L. Zhang X. Rosario A. M. (New York: Wiley), 221–242.

[B25] David M. (2015). The correspondence theory of truth. Available at: https://plato.stanford.edu/entries/truth-correspondence/ (Accessed March 18, 2022).

[B26] Diakopoulos N. (2020). “Transparency,” in The oxford handbook of ethics of AI. Editors Dubber M. D. Pasquale F. Das S. Diakopoulos N. (. New York: Oxford University Press), 196–213.

[B27] Edwards L. Veale M. (2017). Slave to the algorithm? Why a 'right to an explanation' is probably not the remedy you are looking for. Duke Law Technol. Rev. 16, 18–84. 0.31228/osf.io/97upg

[B28] Federal Anti-Discrimination Agency (2019). Guide to the general equal treatment Act. Berlin: Explanations and Examples.

[B29] Felzmann H. Villaronga E. F. Lutz C. Tamò-Larrieux A. (2019). Transparency you can trust: Transparency requirements for artificial intelligence between legal norms and contextual concerns. Big Data & Soc. 6 (1), 205395171986054. 10.1177/2053951719860542

[B30] Fjeld J. Achten N. Hilligoss H. Nagy A. C. Srikumar M. (2020). Principled artificial intelligence: Mapping consensus in ethical and rights-based approaches to principles for AI. Cambridge: Berkman Klein Center for Internet and Society.

[B31] Floridi L. (2017). Infraethics-on the conditions of possibility of morality. Philos. Technol. 30 (30), 391–394. 10.1007/s13347-017-0291-1

[B32] Foucault M. (1972). The archaeology of knowledge: And the discourse on language. New York: Pantheon Books.

[B34] Gitelman L. (2013). Raw data is an oxymoron. Cambridge: MIT Press.

[B35] Goswami S. (2020). “Deep learning – a state-of-the-art approach to artificial intelligence,” in Deep learning: Research and applications. Editors Bhattacharyya S. Snasel V. Hassanien A. E. Saha S. Tripathy B. K. (Berlin, Boston: Walter de Gruyter), 1–19.

[B87] Hague D. C. (2019). Benefits, Pitfalls, and Potential Bias in Health Care AI. North Carol. Med. J. 80, 219–223. 10.18043/ncm.80.4.219 31278181

[B36] Halsema A. (2005). “Reflexionen über Identität in einer multikulturellen Gesellschaft: Ein Dialog zwischen Ricoeur, Irigaray und Butler,” in Feministische Phänomenologie und Hermeneutik. Editors Stoller S. Vasterling V. Fisher L. (Würzburg), 208–234.

[B37] Hasselbalch G. (2021). Data ethics of power. A human approach in the big data and AI era. Massachusetts: Edward Elgar Publishing Inc.

[B38] Hatzenbuehler M. L. Pachankis J. E. (2021). “Sexual and gender minority health disparities: Concepts, methods, and future directions,” in The science of health disparities research. Editors Dankwa-Mullan I. (New York: Wiley), 429–444.

[B39] High-Level Expert Group on Artificial Intelligence (2019). Ethics guidelines for trustworthy AI. Brüssel: European Commission..

[B40] Honneth A. (2003). Unsichtbarkeit: Stationen einer Theorie der Intersubjektivität. Frankfurt am Main: Suhrkamp.

[B41] Hummel P. Braun M. Augsberg S. von Ulmenstein U. Dabrock P. (2021a). Datensouveränität: Governance-Ansätze für den Gesundheitsbereich. Wiesbaden: Springer.

[B89] Huston P. Edge V. L. Bernier E. (2019). Reaping the benefits of Open Data in public health. In: Canada Commun. Dis. Rep. 45, 252–256. 10.14745/ccdr.v45i10a01 PMC6781855 31647060

[B43] Hummel P. Braun M. Tretter M. Dabrock P. (2021b). Data sovereignty: A review. Big Data & Soc. 8, 205395172098201. 10.1177/2053951720982012

[B44] Hwang B. You J. Vaessen T. Myin-Germeys I. Park C. Zhang B.-T. (2018). Deep ECGNet: An optimal deep learning framework for monitoring mental stress using ultra short-term ECG signals. Telemed. J. E. Health. 24 (10), 753–772. 10.1089/tmj.2017.0250 29420125

[B45] Iqbal S. Tariq M. Ayesha H. Ayesha N. (2021). “AI technologies in health-care applications,” in Artificial intelligence and internet of things. Applications in smart healthcare. Editor Goyal L. M. (London: CRC Press), 3–44.

[B46] Jasanoff S. (2016). The ethics of invention. Technology and the human future. New York: W. W. Norton & Company.

[B47] Jobin A. Ienca M. Vayena E. (2019). The global landscape of AI ethics guidelines. Nat. Mach. Intell. 1 (9), 389–399. 10.1038/s42256-019-0088-2

[B48] Jüngel E. (2010). Gott als geheimnis der Welt. Tübingen: Mohr Siebeck.

[B49] Kashif M. Rehman A. Sadad T. Mehmood Z. (2021). “Breast cancer detection and diagnostic with convolutional neural networks,” in Artificial intelligence and internet of things. Applications in smart healthcare. Editor Goyal L. M. (London: CRC Press), 65–84.

[B50] Kilian P. (2013). Unsichtbare Sichtbarkeit. Michel Foucault und die Transparenz. Available at: https://blog.genealogy-critique.net/essays/19/unsichtbare-sichtbarkeit (Accessed March 18, 2022).

[B51] Latour B. (2000). Die Hoffnung der Pandora. Untersuchungen zur Wirklichkeit der Wissenschaft. Aus dem Englischen von Gustav Roßler. Frankfurt am Main: Suhrkamp.

[B52] Lee E. E. Torous J. de Choudhury M. Depp C. A. Graham S. A. Kim H.-C. (2021). Artificial intelligence for mental health care: Clinical applications, barriers, facilitators, and artificial wisdom. Biol. Psychiatry. Cogn. Neurosci. Neuroimaging 6 (9), 856–864. 10.1016/j.bpsc.2021.02.001 33571718 PMC8349367

[B53] Leslie D. Mazumder A. Peppin A. Wolters M. K. Hagerty A. (2021). Does "AI" stand for augmenting inequality in the era of coCovid-19ealthcare? BMJ 372, n304. 10.1136/bmj.n304 33722847 PMC7958301

[B54] Lima G. Grgić-Hlača N. Jeong J. K. Cha M. (2022). The Conflict Between Explainable and Accountable Decision-Making Algorithms. Available at: https://arxiv.org/abs/2205.05306 (Accessed June 3, 2022).

[B55] Lu X. H. Mamiya H. Vybihal J. Ma Y. Buckeridge D. (2020). “Guiding public health policy by using grocery transaction data to predict demand for unhealthy beverages,” in Explainable AI in healthcare and medicine building a culture of transparency and accountability. Editor Shaban-Nejad A. (New York: Springer), 169–176.

[B56] Lundberg S. Lee S-I. (2017). A unified approach to interpreting model predictions. Available at: https://arxiv.org/abs/1705.07874 (Accessed May 31, 2022).

[B57] Lupton D. (2015). “Donna Haraway: The digital cyborg assemblage and the new digital health technologies,” in The palgrave handbook of social theory in health, illness and medicine. Editor Collyer F. (New York: Springer), 567–581.

[B58] Mann M. Matzner T. (2019). Challenging algorithmic profiling: The limits of data protection and anti-discrimination in responding to emergent discrimination. Big Data & Soc. 6, 205395171989580. 10.1177/2053951719895805

[B59] Maschewski F. Nosthoff A.-V. (2021). Überwachungskapitalistische Biopolitik: Big Tech und die Regierung der Körper. Z. für Politikwiss. 32 10.1007/s41358-021-00309-9

[B60] Mayer-Schönberger V. (2009). Delete: The virtue of forgetting in the digital age. Oxford: Princeton University Press.

[B61] Mentzel H.-J. (2021). [Artificial intelligence in image evaluation and diagnosis]. Monatsschr. Kinderheilkd. 169 (8), 694–704. 10.1007/s00112-021-01230-9 34230692 PMC8250551

[B62] Meunier A. Gray J. Ricci D. (2021). A new AI lexicon: Algorithm trouble. Troublesome encounters with algorithms that go beyond computational processes. Available at: https://medium.com/a-new-ai-lexicon/a-new-ai-lexicon-algorithm-trouble-50312d985216 (Accessed June 5, 2022).

[B63] Mittelstadt B. Allo P. Taddeo M. Wachter S. Floridi L. (2016). The ethics of algorithms: Mapping the debate. Big Data & Society 1–21. 10.1177/2053951716679679

[B64] Norori N. Hu Q. Aellen F. M. Faraci F. D. Tzovara A. (2021). Addressing bias in big data and AI for health care: A call for open science. Patterns 2 (10), 100347–100349. 10.1016/j.patter.2021.100347 34693373 PMC8515002

[B65] Norris C. M. Yip C. Y. Y. Nerenberg K. A. Clavel M.-A. Pacheco C. Foulds H. J. A. (2020). State of the science in women's cardiovascular disease: A Canadian perspective on the influence of sex and gender. J. Am. Heart Assoc. 9, e015634. 10.1161/JAHA.119.015634 32063119 PMC7070224

[B66] Obermeyer Z. Powers B. Vogeli C. Mullainathan S. (2019). Dissecting racial bias in an algorithm used to manage the health of populations. Science 366, 6464447–6464453. 10.1126/science.aax2342 31649194

[B67] Oskooei A. Chau S. M. Weiss J. Sridhar A. Martínez M. R. Michel B. (2021). “DeStress: Deep learning for unsupervised identification of mental stress in firefighters from heart-rate variability (HRV) data,” in Explainable AI in healthcare and medicine building a culture of transparency and accountability. Editors Shaban-Nejad A. Michalowski M. Buckeridge D. L. (Cham: Springer Nature), 93–105.

[B68] Pasquale F. (2015). Black box society. Cambridge: Harvard University Press.

[B69] Prabhakaran V. Martin D. (2020). Participatory Machine Learning Using Community-Based System Dynamics. Health Hum. Rights 22 (2), 71–74. 33390696 PMC7762892

[B70] Prainsack B. (2017). Personalized medicine: Empowered patients in the 21st century? New York: Wiley.

[B71] Prince A. E. R. Schwarcz D. (2020). Proxy discrimination in the age of artificial intelligence and big data. Iowa Law Review. Available at: https://heinonline.org/hol-cgi-bin/get_pdf.cgi?handle=hein.journals/ilr105§ion=35 (Accessed June 13, 2022).

[B72] Röösli E. Rice B. Hernandez-Boussard T. (2021). Bias at warp speed: How AI may contribute to the disparities gap in the time of COVID-19. J. Am. Med. Inf. Assoc. 28 (1), 190–192. 10.1093/jamia/ocaa210 PMC7454645 32805004

[B73] Ruf B. Detyniecki M. (2021). Towards the right kind of fairness in AI. Available at: http://arxiv.org/pdf/2102.08453v7 (Accessed March 18, 2022).

[B75] Schütz F. Heidingsfelder M. L. Schraudner M. (2019). Co-Shaping the future in quadruple helix innovation systems: Uncovering public preferences toward participatory research and innovation. She Ji J. Des. Econ. Innovation 5 (2), 128–146. 10.1016/j.sheji.2019.04.002

[B76] Seyyed-Kalantari L. Zhang H. McDermott M. B. A. Chen I. Y. Ghassemi M. (2021). Underdiagnosis bias of artificial intelligence algorithms applied to chest radiographs in under-served patient populations. Nat. Med. 27 (12), 2176–2182. 10.1038/s41591-021-01595-0 34893776 PMC8674135

[B77] Stark H. (2014). Judith Butler’s post-Hegelian ethics and the problem with recognition. Fem. Theory 15 (1), 89–100. 10.1177/1464700113512738

[B78] Strotbaum V. Reiß B. (2017). “„Apps im Gesundheitswesen – echter medizinischer Nutzen oder der Weg zum gläsernen Patienten,” in E-Health-Ökonomie. Editors Müller-Mielitz T. Lux S. (Wiesbaden: Springer Fachmedien Wiesbaden), 359–382.

[B90] Turek H. (2020). Open algorithms: Experiences from France, the Netherlands and New Zealand (Open Algorithms Blog Series). Available at: https://www.opengovpartnership.org/stories/open-algorithms-experiences-from-france-the-netherlands-and-new-zealand/ Accessed Jully 24, 2022

[B79] Uddin M. Z. Dysthe K. K. Følstad A. Brandtzaeg P. B. (2022). Deep learning for prediction of depressive symptoms in a large textual dataset. Neural comput. Appl. 34 (1), 721–744. 10.1007/s00521-021-06426-4

[B91] UNESCO (2022). Recommendation on the ethics of artificial intelligence. Paris.

[B88] Viola L. A. Laidler P. (2021). Trust and transparency in an age of surveillance. London: Routledge.

[B80] Véliz C. (2020). Privacy is power: Why and how you should take back control of your data. London: Penguin Books.

[B81] Wachter S. Mittelstadt B. (2019). A right to reasonable inferences: Re-thinking data protection law in the age of big data and AI. Columbia Bus. Law Rev. 2, 494–630. 10.7916/cblr.v2019i2.3424

[B82] Wachter S. (2022). The theory of artificial immutability: Protecting algorithmic groups under anti-discrimination law. Tulane Law Review 97. 10.2139/ssrn.4099100 Available at: https://papers.ssrn.com/sol3/papers.cfm?abstract_id=4099100 Accessed Jully 24, 2022.

[B83] Walter M. (2018). The voice of Indigenous data. Beyond the markers of disadvantage. Available at: https://griffithreview.com/articles/voice-indigenous-databeyond- (Accessed May 31, 2022).

[B84] WHO (2021). Ethics and governance of artificial intelligence for health. Available at: https://www.who.int/publications/i/item/9789240029200 (Accessed March 18, 2022).

[B85] Zerilli J. Bhatt U. Weller A. (2022). How transparency modulates trust in artificial intelligence. Patterns 3 (4), 1–10. 10.1016/j.patter.2022.100455 PMC9023880 35465233

[B86] Zerilli J. Dahner J. Maclaurin J. Gavaghan C. Knott A. Liddicoat J. (2021). A citizen's guide to artificial intelligence. Cambridge: MIT Press.

